# Dynamic Measurement for the Diameter of A Train Wheel Based on Structured-Light Vision

**DOI:** 10.3390/s16040564

**Published:** 2016-04-20

**Authors:** Zheng Gong, Junhua Sun, Guangjun Zhang

**Affiliations:** Key Laboratory of Precision Opto-Mechatronics Technology, Ministry of Education, Beihang University, No. 37 Xueyuan Road, Haidian District, 100191 Beijing, China; gongz@buaa.edu.cn (Z.G.); sjh@buaa.edu.cn (J.S.)

**Keywords:** train wheel diameter, dynamic measurement, structured-light vision, machine vision

## Abstract

Wheels are very important for the safety of a train. The diameter of the wheel is a significant parameter that needs regular inspection. Traditional methods only use the contact points of the wheel tread to fit the rolling round. However, the wheel tread is easily influenced by peeling or scraping. Meanwhile, the circle fitting algorithm is sensitive to noise when only three points are used. This paper proposes a dynamic measurement method based on structured-light vision. The axle of the wheelset and the tread are both employed. The center of the rolling round is determined by the axle rather than the tread only. Then, the diameter is calculated using the center and the contact points together. Simulations are performed to help design the layout of the sensors, and the influences of different noise sources are also analyzed. Static and field experiments are both performed, and the results show it to be quite stable and accurate.

## 1. Introduction

In a train, wheelsets bear all static and dynamic loads. The wear, peeling and scraping of wheel treads can cause the change of the diameter and out-of-roundness. The diameter differences between the wheels of one wheelset, one bogie, one car and one train should be controlled under a safe limitation; otherwise, it will accelerate the wear of wheels and bring harm to the train operation.

Nowadays, the measurement of the wheel diameter can be divided into two categories: static measurement and dynamic measurement. Static measurement is usually performed during the maintenance of wheels using special mechanical calipers or automatic measurement devices [1,2]. The mechanical caliper is mostly based on the theory of the maximum distance or the theory of arch-height and chord-length. The resolution of the caliper is usually 0.1 mm, and the skill of the operator can influence the result seriously. The automatic measurement devices use mechanical methods or optical methods to realize the noncontact measurement. Feng *et al.* [1] utilized five sets of parallelogram mechanisms together with laser transducers to realize the diameter measurement. Wu *et al.* [2] utilized the laser displacement sensor (LDS) and the charge coupled device (CCD) to measure the diameter. These methods have high precision. However, the wheel must be disassembled from the bogie and rotate on a fixed axle of the device for the diameter measurement. It takes much time and can only be performed in train yards or in train shops. Dynamic measurement is performed when a train passes the measurement system that is mounted on the rail track. LDS [3,4,5,6] and structured-light vision [7,8,9,10,11] are the main methods utilized in the dynamic measurement. The LDS methods measure the distance from the contact point to the sensor when the wheel arrives at a predetermined position in which a triangular geometry relationship can be established between the distance and the diameter. The LDS methods need a high precision mechanism to guarantee the triangular geometry, and the predetermined positon is very important for the measurement accuracy. Mian *et al.* [3] utilized three precise wheel location detectors to measure the positon, and the diameter was calculated through measurements four times. Zhang *et al.* [4] utilized one eddy sensor to measure the position and two LDSs to measure the diameter. In order to improve the accuracy of the position, Gao *et al.* [5] utilized two eddy sensors, and the position error was 0.02 mm. Wu *et al.* [6] utilized the LDS and high-speed CCD to realize the diameter measurement, and the accuracy was within 1.2 mm, while the train was running in a limited speed less than 80 km/h. The structured-light vision methods measure the profiles of the wheel tread, and the contact points are determined from those profiles. The rolling round is then fitted. The structured-light vision methods do not need a high precision mechanism, but multiple sensors or repeated measurements are needed to achieve at least three contact points. The 3D reconstruction of the sensors and the image processing are the key techniques of these methods. Sanchez-Revuelta *et al.* [7] utilized a line laser that was parallel to the rolling round. However, a tailor-made steel slab was needed to support the wheel. Gao *et al.* [8] used a line laser to achieve multiple profiles of the wheel tread via repeated measurements, and the velocity of the wheel must be measured precisely. There are also several systems [9,10,11] produced by some corporations. These systems use structured-light sensors to achieve multiple profiles of the wheel tread from the front and the back of the wheel. Then, the contact points on the multiple profiles are determined and are used to fit the rolling round. However, the circle fitting algorithm is very sensitive to noise when only a small number of points are used. What is more, the areas of the wheel, which can be measured by the laser, are very limited because many mechanical parts are around the wheel tread. Therefore, only three contact points can be used in most systems, which is not robust. Our previous work [12] expanded the distribution of the contact points by measurements twice. The cycloid curve constraint was used to integrate the contact points measured at two different positions. However, a high precision wheel location was needed to guarantee the measurement accuracy. Additionally, it was sensitive to the vibrations on the rock track-bed.

In order to improve the robustness, we propose a method for the diameter measurement using the axle of the wheelset instead of the wheel tread only. The axle is measured by one structured-light sensor, and the wheel tread is measured by three structured-light sensors. The center of the rolling round is determined by the axle. The radius of the rolling round is determined by the center and the contact points on the wheel tread. Some key technologies, including the 3D reconstruction of the structured-light vision and the image processing, are described. Simulations are performed to analyze the influences of different noise sources and to help with designing the layout of the sensors. The static experiment was performed in the laboratory, and the field experiment was performed on a railway, where the train was running at a high speed of about 80 km/h. The results showed it to be quite accurate and robust.

This paper is organized as follows: Section 2 describes the measurement model and method, including the structured-light model, the 3D reconstruction, the image processing and the calculation of the diameter. Section 3 describes the simulations with respect to the 3D reconstruction noise, the deformation and the geometrical error of the axle. According to the simulations, the most appropriate layout of the sensors is determined. Section 4 describes the static experiment and the field experiment, and the conclusion is presented in Section 5.

## 2. Measurement Model and Method

**Figure 1:**
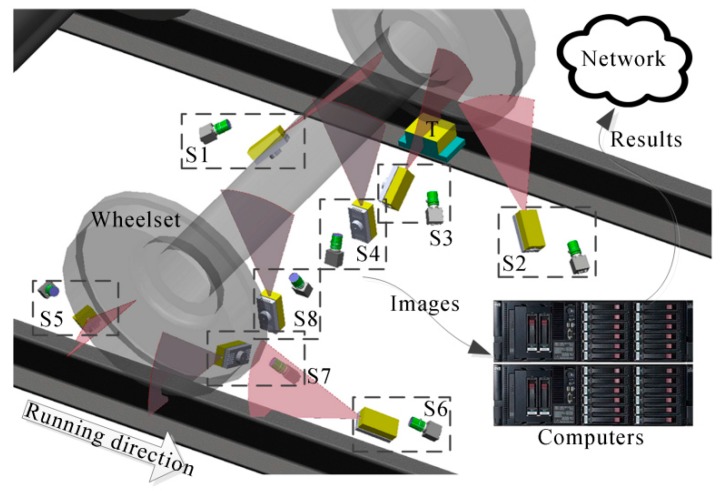
Measurement system structure. S1–S8: Structured-light sensors; T: electromagnetic sensor.

The wheel is measured by four structured-light sensors, three of which measure the wheel tread, and one measures the axle of the wheelset. We install an electromagnetic sensor for the detection of the wheel on the rail track. When a wheel arrives at the right above the sensor, it will send a trigger signal to make all of the structured-light sensors project line lasers and capture the laser images. The laser images are transmitted to the computers for image processing and 3D reconstruction. Then, the contact points and the center of the rolling round are determined, and the diameter is calculated. Figure 1 shows the structure of the system. Eight structured light sensors are needed to measure both wheels of a wheelset. The measurement results are uploaded onto the network for long-distance use, which is the requirement of being unattended. Without loss of generality, only four sensors for one side of the wheelset are discussed in the following, while the other four are totally symmetric.

### 2.1. Structured-Light Vision Model

**Figure 2:**
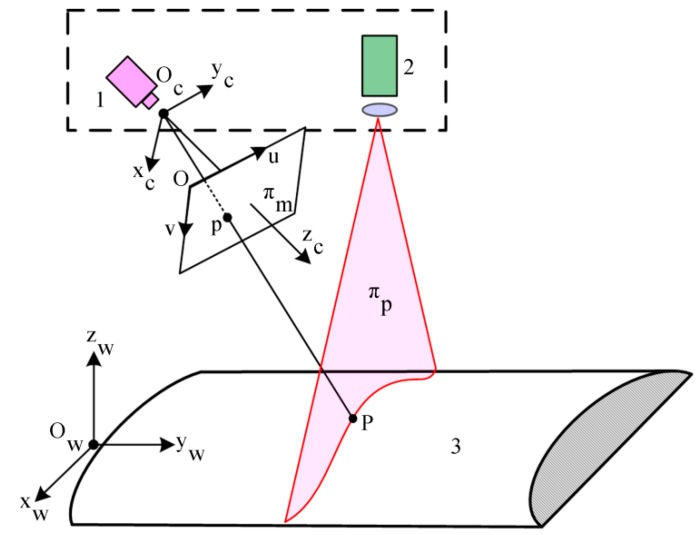
Structured-light vision model. (1) Camera; (2) laser projector; (3) measured object.

The structured-light sensor is composed of one line laser projector and one camera. The camera captures the image of the laser profile that is the intersection line of the laser plane (as $\pi_{P}$ shown in Figure 2) and the measured object. The task of structured-light vision is to compute the 3D coordinates of the laser profile. The measurement 3D coordinate frame is usually established in the camera, which is the camera coordinate frame (CCF) O_c_-x_c_y_c_z_c_, as shown in Figure 2. The axis O_c_z_c_ is the optical axis of the lens, and O_c_ is the optical center of the lens. The axes O_c_x_c_ and O_c_y_c_ are the horizontal line and the vertical line of the imaging plane, respectively. The image coordinate frame (ICF) is established on the image captured by the camera, which is O-uv, as shown in Figure 2. The units of ICF are in pixels. In a measurement system of multiple sensors, a world coordinate frame (WCF) should be established for the unification of different sensors. Figure 2 shows the schema of the structured-light vison model.

Let ***P*** be a point on the intersection line of the laser plane and the measured object. Its homogeneous coordinate is $\tilde{P}={\left[x_{w},y_{w},z_{w},1\right]}^{T}$, and its projection point on the image is $\tilde{p}={\left[u,v,1\right]}^{T}$. According to the pinhole camera model, we have:

(1) $$\rho \tilde{p}=A[\begin{array}{cc} R & t \end{array}]\tilde{P}$$

where *ρ* is the scale factor. ***R*** and ***t***, called the extrinsic parameters, are the rotation matrix and translation vector from WCF to CCF, respectively. ***A*** is the matrix of camera intrinsic parameters, which can be written as:

(2) $$A=\left[\begin{array}{ccc} f_{x} & \gamma & u_{0}\\ 0 & f_{y} & v_{0}\\ 0 & 0 & 1 \end{array}\right]$$

where *f_x_*, *f_y_* are the scale coefficient with respect to the O-u and O-v axis of ICF. γ is the skew coefficient defining the angle between the u and v pixel axis. (*u_0_,v_0_*)^T^ is the principal point.

However, there always exists distortion in lenses, especially radial distortion. The coordinate of the point on the image should be undistorted for use in the pinhole model. In this paper, we consider only the first two terms of the radial distortion, which make the main contribution. Let $p={\left[x_{u},y_{u}\right]}^{T}$ and $p_{d}={\left[x_{d},y_{d}\right]}^{T}$ be the distortion-free and the normalized distorted image coordinates, respectively. We have:

(3) $$\left(\begin{array}{c} x_{d}\\ y_{d} \end{array}\right)=\{\begin{array}{c} \left(1+k_{1}\left(x_{u}^{2}+y_{u}^{2}\right)+k_{2}{\left(x_{u}^{2}+y_{u}^{2}\right)}^{2}\right)x_{u}\\ \left(1+k_{1}\left(x_{u}^{2}+y_{u}^{2}\right)+k_{2}{\left(x_{u}^{2}+y_{u}^{2}\right)}^{2}\right)y_{u} \end{array}$$

where *k_1_* and *k_2_* are the coefficients of the radial distortion.

Using the above model, we can determine a ray on which the point ***P*** lies, and its equation in CCF can be calculated from Equations (1) and (3) as:

(4) $$\left\{ \begin{array}{l} x_{c}=((x_{u}-u_{0})z_{c}-\gamma y_{c})/f_{x}\\ y_{c}=(y_{u}-v_{0})z_{c}/f_{y} \end{array}\right.$$

where (*x_c_, y_c_, z_c_*) is the coordinate of ***P*** in CCF, which satisfies ${\left[x_{c},y_{c},z_{c}\right]}^{T}=R\cdot {\left[x_{w},y_{w},z_{w}\right]}^{T}+t$. (*x_u_, y_u_*) can be determined by solving the nonlinear Equation (3) using the Levenberg–Marquardt method.

The laser plane is used for the calculation of the 3D coordinate of ***P***. Its equation in CCF can be written as:

(5) $$\pi_{l}:a_{l}x_{c}+b_{l}y_{c}+c_{l}z_{c}+d_{l}=0$$

Then, we can obtain the unique solution of ***P*** via Equations (4) and (5).

### 2.2. Configuration of the Structured-Light Sensor

For the structured-light sensor, there are several key configurations, including the focal length of the lens, the resolution of the camera, the power and the line width of the laser. As shown in Figure 1, the sensors are installed on the railway near the rail track. The distance from the sensor to the wheel tread is about 400 mm, and the field of view (FOV) for the wheel tread measurement is about 220 mm × 180 mm. We choose that the resolution of the camera be 1360 × 1024 pixels, so the resolution of one pixel is about 0.18 mm/pixel. In order to cover the whole FOV, we choose that the size of CCD be 8.8 mm × 6.6 mm, and the focal length of the lens is 12 mm. Equally, the measurement distance from the sensor to the axle of the wheelset is about 1000 mm, and the FOV for the axle of the wheelset is about 350 mm × 350 mm. We choose the same resolution of the camera, so the resolution of one pixel is about 0.25 mm/pixel. We choose that the size of CCD be also 8.8 mm × 6.6 mm, and the focal length of the lens is 17 mm. Besides the camera and the lens, the laser is also an important component. In the railway application, the wheel tread suffers high frequency friction with the rail track, which makes the surface of the wheel tread have high specular reflection. In order to guarantee that the camera can achieve enough light intensity of the reflected laser, the power of the laser should be strong. Therefore, we choose an 808-nm wavelength laser with the power of 4 W, and the line width of the laser is 0.5 mm for a clear and thin laser stripe on the image.

### 2.3. Calibration of the Structured-Light Vision Model

**Figure 3:**
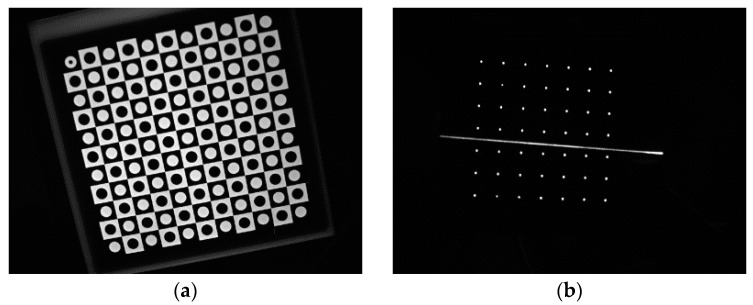
The images of the planar targets. (**a**) The planar target image for the intrinsic parameters’ calibration. The target contains 10 × 10 checkers at the interval of 10 mm, and the precision is 0.02 mm; (**b**) The planar target image for the structured light calibration. The target contains a 7 × 7 dot matrix at the interval of 12 mm, and the precision is 0.02 mm.

The ***A***, ***R***, ***t***, ***k_1_***, ***k_2_*** and $\pi_{l}$ constitute the parameters of the entire structured-light vision model. The calibration for these parameters is very important. In short distance measurement, the calibration is usually performed utilizing high precision targets. 1D targets, planar targets and 3D targets are the main target types. The 3D target is the most simple, but the cost is very high, especially in the measurement for a large range. The 1D target is easily made. However, the features on the 1D target are usually not many, so the calibration precision is not very high. The planar target has both advantages of low cost and abundant features. We design two planar targets for the intrinsic parameters calibration and the structured light calibration, respectively. The camera calibration method proposed by Zhang [13] and the structured-light calibration method by Sun *et al.* [14] are utilized in our practice. These methods are flexible for field use, and the results are precise. The images of the targets are shown in Figure 3.

**Figure 4:**
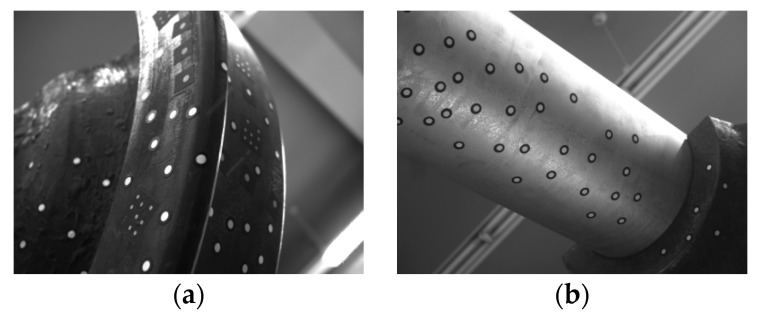
The images for the global calibration. (**a**) The target image of the camera for the wheel tread measurement; (**b**) the target image of the camera for the wheelset axle measurement.

What is more, the four sensors for a wheel should be unified to one WCF. We choose one CCF as the WCF, so a global calibration should be performed to determine the rotation and translation matrix from other CCFs to the WCF. The cameras of the system are non-overlapping, and the distribution is wide, which makes the global calibration difficult. A large 3D control field is usually used in the global calibration. However, a high precision and big control field is expensive and not flexible. It is usually established in an indoor environment, just like a laboratory, it cannot be used in the field railway. Some flexible methods were also proposed [15,16,17]. They used an intermediary to unify any pair of the sensors of the system one by one. However, calibration error could be transmitted and accumulated between the sensors. The sensors of our system have different FOVs and different focal lengths, so it is not easy to design or place the intermediary. We propose a flexible global calibration method for non-overlapping multi-cameras with the help of a handy 3D scanner. We use a general wheelset to build the 3D target that is also the measurement target of the vision system itself. We paste the high reflective circular feature points on the surface of the wheelset, which is exactly in the FOV of the cameras. The 3D positions of the points can be measured by the scanner with a precision of 0.05 mm. Figure 4 shows the images of the feature points. The work of building the 3D target can be performed everywhere, provided the 3D scanner and a general measurement target, so it is very flexible for the field application, and the cost is very low.

We perform the calibration of the entire vision system using the above techniques. The calibration errors are as follows: the re-projection error of the intrinsic parameters calibration is about 0.03 pixels; the RMS error of fitting the laser plane is about 0.05 mm; and the re-projection error of the global calibration is about 0.3 pixels. According to the resolution of the camera (that is about 0.25 mm/pixel, which is described in Section 2.2), we can estimate that the 3D reconstruction error of the vision system is about 0.1 mm. This error is the intrinsic error of the vision system when the calibration is finished. However, there are some other errors caused by the vibration, ambient lights, the motion of wheels and the noise reflected by the surface of the wheel tread. These errors are usually much greater than the calibration error, so they are the main factors influencing the measurement result.

### 2.4. Image Processing

**Figure 5:**
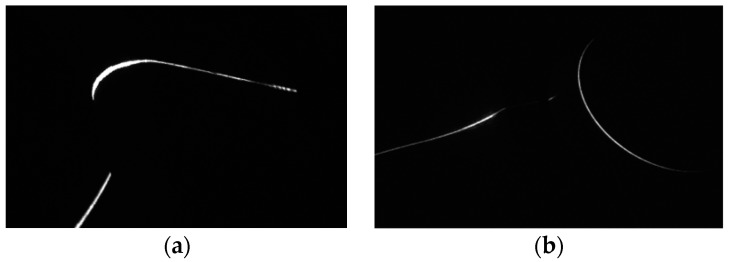
Image of the laser stripe. (**a**) Image of the wheel tread; (**b**) image of the axle of the wheelset.

Image processing is very important for the calibration and the 3D reconstruction of the structured-light vision. The accuracy of the center of the laser stripe greatly influences the measurement result. Meanwhile, a thin and strong laser stripe is also a good precondition of the accurate image processing. However, the surface of the wheel tread is highly specularly reflective, and the measurement system is installed on an open field. Ambient lights and noise lights will bring in strong disturbance. In order to enhance the intensity of the laser and to weaken the ambient lights, we use the high power laser projector and the narrow-band optical filter. In this condition, we achieve the laser stripe image as shown in Figure 5. It is noticed that the intensity and the width of the light is not equal along with the strip. The width varies from about 3 pixels to 15 pixels, and the gray level varies from about 40 to 255.

**Figure 6:**
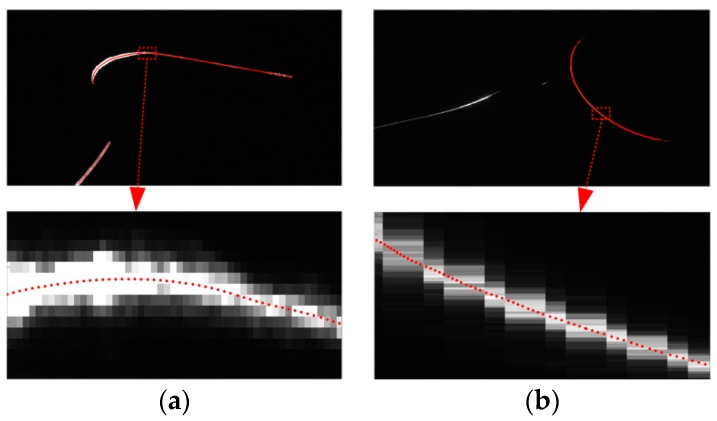
The image processing result of Figure 5. (**a**) The result of Figure 5a; (**b**) the result of Figure 5b.

Steger [18] proposed a subpixel center extraction method for curvilinear structures. It detected the maximum of the second derivative of the gray level perpendicular to the laser stripe. It could weaken the influence of noises and reflect lights by using the Gaussian filter. It could adapt any width of light stripe provided the proper parameters of the Gaussian template. In order to improve the processing speed, Chen *et al.* [19] proposed a revised method that used the direction-guided algorithm. It linked the subpixel centers along the local stripe direction and removed the unwanted pixels in the perpendicular direction. Therefore, it had a remarkable speedup and avoided the false light stripe that we did not want. We use Chen’s method to perform the image processing. The image processing result of Figure 5 is shown in Figure 6. The accuracy of the center extraction is 0.1 pixels. It can be seen in Figure 6 that the method is effective for the changing width and the changing gray level of the light stripe. In Figure 6b, there is also a false laser stripe that is not processed in the left part of the image.

### 2.5. Wheel Tread Profile and Wheel Diameter

**Figure 7:**
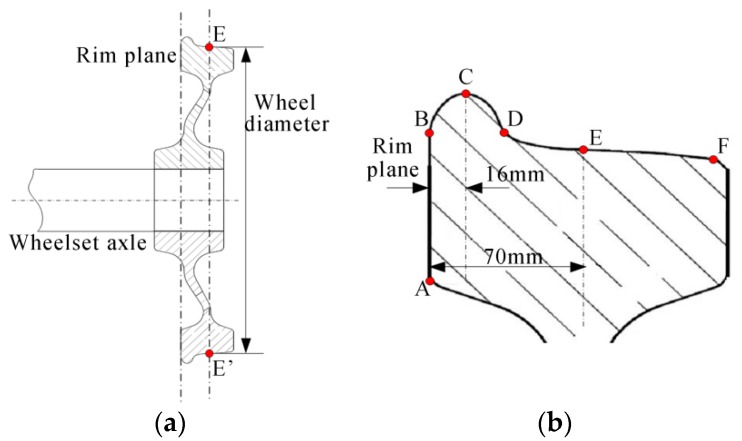
The profiles of the wheelset and tread. (**a**) The profile of half wheelset; (**b**) the profile of wheel tread; AB: the wheel rim plane; BD: the wheel flange; C: the flange vertex; DF: the wheel tread; E: the contact point.

The profiles of the wheelset and the wheel tread are shown in Figure 7. There is a contact point on the wheel tread that is contacted with the rail track according to its design. This point is usually a 70-mm distance to the wheel rim plane for most trains. All of the contact points around the wheel constitute a circle that is called the rolling round. The wheel diameter is defined as the diameter of the rolling round.

### 2.6. Calculation of the Diameter

The flange vertex, the contact point and the axis of the wheelset axle are employed to determine the wheel diameter. Firstly, the sensors S1-S3 in Figure 1 obtain the profile of the wheel tread. Three flange vertexes, three rim plane segments and two contact points are determined from the profile. Secondly, S4 obtains the profile of the cross-section of the wheelset axle; then, the circle is fitted using the profile, and the center that is on the axis of the wheelset axle is determined. Finally, we can determine the wheel center that is the intersection of the rim plane and the axis, and then, the diameter can be calculated.

#### 2.6.1. Determine the Rim Plane, the flange Vertex and the Contact Point

**Figure 8:**
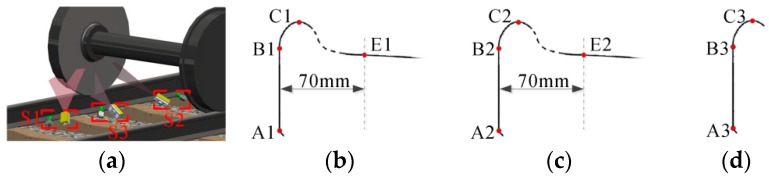
The wheel tread measurement. (**a**) The sensors for tread measurement; (**b**) the result of S1; (**c**) the result of S2; (**d**) the result of S3.

The sensors for the wheel tread measurement are shown in Figure 8a, and the results are shown in Figure 8b–d. S1 and S2 obtain the profiles of the rim plane, the flange and the tread. S3 can only obtain the rim plane and the flange due to the covering of the rail track.

The points of the profile of each sensor are in the same laser plane, so we can convert the 3D coordinates of the profile to the 2D coordinate frame that is established on the laser plane. The 2D coordinate frame is arbitrary on the plane, so the position and pose of the 2D profile is also arbitrary. We need to find the rim plane segment and then rotate the profile to the direction that the rim plane segment is vertical, as shown in Figure 8b–d. Since the points of the profile are linked in the direction that is from the end point of the rim plane to the end point of the tread in the image processing, we can search the straight line part as the rim plane segment from the beginning. The searching method is as follows:

(1) Set *q0* as the start point of the profile.
(2) In order to avoid the noise and the influence of the wheel spoke, we find the first point q1 satisfying ‖q0−q1‖>3mm as the start of the straight line.
(3) Generally, the rim plane segment is always longer than 25 mm. We can continue to search the profile, and add all of the points *qi* satisfying: ‖qi−q1‖<15mm into the straight line part. Then, the initial straight line can be fitted using these points. The linear least-squares algorithm can be used. Because these points must be a part of the rim plane, the fitting error should be very small.
(4) Add the next point into the straight line part and fit the line again. If the max distance from all of the points to the fitted line is smaller than 0.2 mm, then the point can be added to the straight line part; otherwise, the point should be removed.
(5) Repeat Step (4) with the rest points one by one until the whole profile is searched. Then, we can obtain the rim plane segment and the line equation.

We need to rotate the profile according to the line equation, so that the rim plane segment can be parallel to the y-axis of the 2D coordinate frame on the laser plane, as shown in Figure 8b–d. Then, the flange vertex ***C*** is the highest point of the profile along the y-axis, and the contact point ***E*** can also be determined by the distance to the rim plane segment.

With the results of S1–S3, three rim plane segments can be determined. Using the 3D coordinates in the WCF, we can obtain the equation of the wheel rim plane by fitting all of the rim plane segments:

(6) $$\pi_{rim}:\;a_{r}x+b_{r}y+c_{r}z+d_{r}=0$$

#### 2.6.2. Determine the Axis of the Wheel Axle

**Figure 9:**
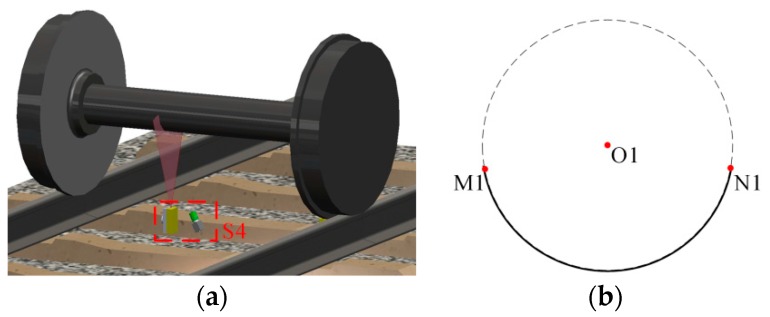
The wheelset axle measurement. (**a**) The sensor for the axle; (**b**) the result of S4.

The sensor for the wheelset axle measurement is shown in Figure 9a, and the result is shown in Figure 9b. S4 can obtain the profile of the cross-section of the wheelset axle.

The same as the wheel tread, the profile needs to be converted to the 2D coordinate frame, and then, a circle is fitted using the 2D profile. The center of the circle is on the axis of the wheelset axle. The 3D coordinate of the center ***O*1** in WCF can be determined reversely. Using the wheel rim plane, we can obtain the equation of the axis in WCF as:

(7) $$L:\;\frac{x-x_{O1}}{a_{r}}=\frac{y-y_{O1}}{b_{r}}=\frac{z-z_{O1}}{c_{r}}$$

where (*x*_*O*1_,*y*_*O*1_,*z*_*O*1_) is the coordinate of ***O1*** and (*a_r_*, *b_r_*, *c_r_*) is the normal vector of the rim plane.

#### 2.6.3. Solve the Wheel Diameter

**Figure 10:**
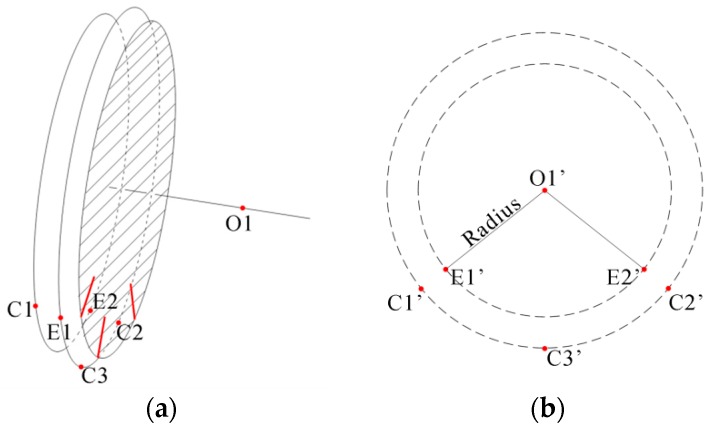
Determine the radius of the wheel. (**a**) The feature points in the 3D coordinate frame; (**b**) the projection of the feature points to the wheel rim plane.

In order to perform the calculation on the wheel rim plane, the three flange vertexes and two contact points should be projected to the wheel rim plane, as shown in Figure 10. The intersection of the axis of the wheelset axle and the wheel rim plane is ***O*1**′.

The projection to the wheel rim plane can be calculated by:

(8) $$P\prime =P+t\times \frac{N^{T}}{\left\|N\right\|}$$

where ***N*** = (*a_r_*, *b_r_*, *c_r_*) is the vector of the axis, ***P***′ is the projection of ***P*** (any one of ***E1***, ***E2***, ***C1*** to ***C3*** or ***O*1**) and *t* is the distance from ***P*** to the wheel rim plane, which can be written as:

(9) $$t=\frac{\left|N\cdot P+d_{r}\right|}{\left||N|\right|}$$

Since the 3D reconstruction noise exists in the measurement, we can use the flange vertexes to optimize the coordinate of ***O*1**′. The maximum likelihood criterion is used against the noise and the objective is that the wheel rim plane center is the most equidistant to ***C1***′ to ***C3***′ and the most near to the initial position of ***O*1**′. Therefore, the objective function can be written as:

(10) $$\min F(O_{o})=\underset{O_{o}}{\min}\left({\left(\left\|O_{o}-C1\prime \right\|-\left\|O_{o}-C2\prime \right\|\right)}^{2}+{\left(\left\|O_{o}-C1\prime \right\|-\left\|O_{o}-C3\prime \right\|\right)}^{2}+{\left\|O_{o}-O1\prime \right\|}^{2}\right)$$

where ***O_o_*** is the optimized wheel center on the rim plane. A nonlinear optimization method, such as Levenberg–Marquardt, can be performed. The wheel diameter *D* can be calculated as:

(11) $$D=\left\|O_{o}-E1\prime \right\|+\left\|O_{o}-E2\prime \right\|$$

## 3. Simulation Analysis

**Figure 11:**
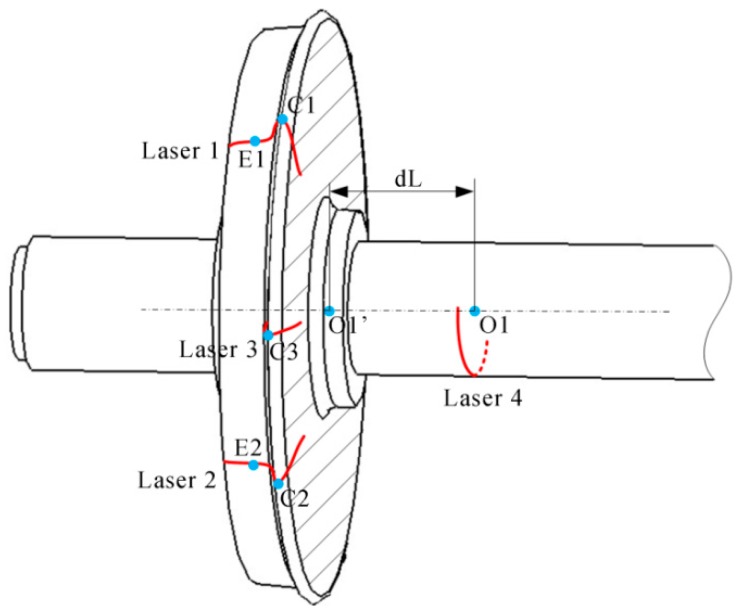
The simulation frame. The diameter of the wheel is set to 840 mm, and dL is the distance from ***O1*** to the wheel rim plane.

The measurement result is mainly affected by three factors: the measurement noise of the structured-light sensors, the deformation of the wheelset and the geometrical error of the wheelset axle. The flange vertex, the contact point, the wheel rim plane and the center of the axle are directly determined by the measurement of the sensors. Since the wheel rim plane is fitted by the three profiles from three sensors, the layout of the profiles is related to the measurement robustness. In the calculation of the center of the wheel, we have made an assumption that the wheelset axle is perpendicular to the wheel rim plane as its design. However, the wheelset axle is slightly curved in actual operation due to the heavy load on the axle. This deformation can affect the actual position of the wheel center on the rim plane. What is more, the geometrical error of the wheelset axle in production also leads to the error of the center position. The position of the laser profile on the axle is related to the center result. Therefore, the simulations with different noises are performed for two purposes. One is to verify whether the configuration of the sensors can satisfy the accuracy requirement, and the other is to determine the layout of the sensors for the best robustness against the noise. The simulation frame is constructed as shown in Figure 11.

### 3.1. The Factor of the Measurement of the Structured-Light Sensors

The resolution of the camera, the calibration of the vision system, the image processing and the vibration are the factors related to the measurement result of the sensors, as described in Section 2.2 and Section 2.3. The resolution of the camera is about 0.25 mm/pixel, and the calibration brings in the intrinsic error, which is usually small. The ambient light and the high reflection on the laser image can affect the image processing seriously. Although the center extraction method can adapt any width and gray level of the light stripe, the actual center is biased due to the reflection. Meanwhile, the wheel tread surface is highly reflective due to the continual friction with the rail track, so the width and strength of the laser stripe has great randomness, which causes the center error to be random, of about 0.5 pixel. The vibration of the vision system can also cause the shift of the laser stripe on the image, and it can change the parameters of the vision model. However, we have reduced the vibration transmitted to the sensors from the rail by the design of the mechanism. In our experience, the vibration can cause about a one-pixel shift of the laser stripe on the image.

**Figure 12:**
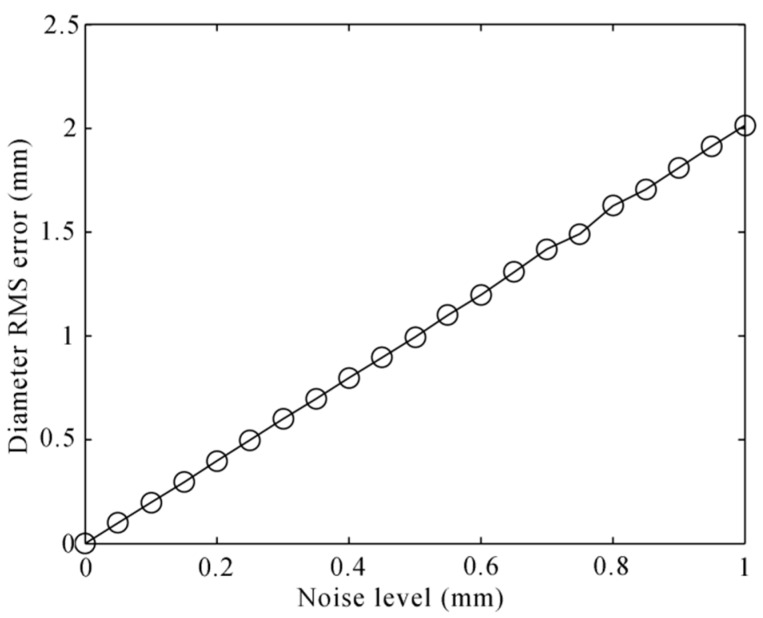
Diameter RMS error to the 3D noise of the structured-light sensors.

The measurement error of structured-light sensors directly reflects in the 3D coordinates of the laser profiles. Without loss of generality, we add the Gaussian noises with a 0 mm mean and standard deviation from 0.0 mm to 1.0 mm at the interval of 0.05 mm to the 3D profiles in WCF. For each noise level, 10,000 independent trials are performed, and the RMS error is computed. As shown in Figure 12, the RMS error is approximately linear, growing with the noise level. The noise level of the structured-light sensors is about 0.2 mm in our analysis. However, sometimes, the wheel tread will have been scraped or exfoliated on the surface, which will lead to a big error of about 1 mm of the contact point.

**Figure 13:**
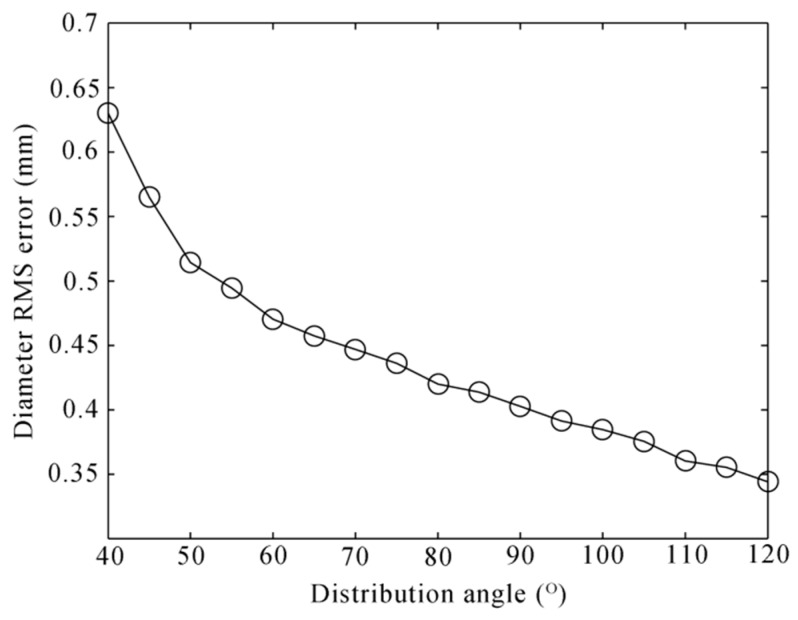
Diameter RMS error to the distribution angle of the lasers on the wheel rim plane.

The wheel rim plane is fitted by three profiles, so the layout of the profiles is important for the fitting result. We change the distribution angle of the lasers on the wheel rim plane from 40° to 120° at the interval of 5°. For each angle, the noise level is set to 0.2 mm, and 10,000 independent trials are performed. The RMS error is decreasing along with the angle increasing, as shown in Figure 13. However, the wide distribution of the laser profiles is hard to achieve, because some attachments, such as the brake shoes, are around the wheel tread, which will block the camera and the laser. In our experience, the angle of 90° is most appropriate, and the simulation RMS error is about 0.4 mm.

### 3.2. The Factor of the Deformation of the Wheelset Axle

**Figure 14:**
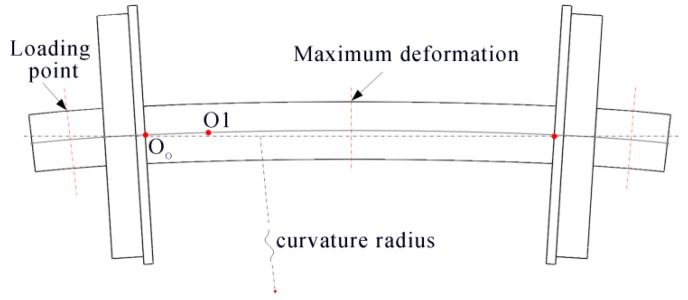
Deformation of the wheelset.

In actual operation, both ends of the wheelset axle bear all of the loads of the vehicle. This will lead to a slight bending of the axle, which makes the axis of the axle not perpendicular to the wheel rim plane. The axle usually bends like an arc curve approximately, as shown in Figure 14.

**Figure 15:**
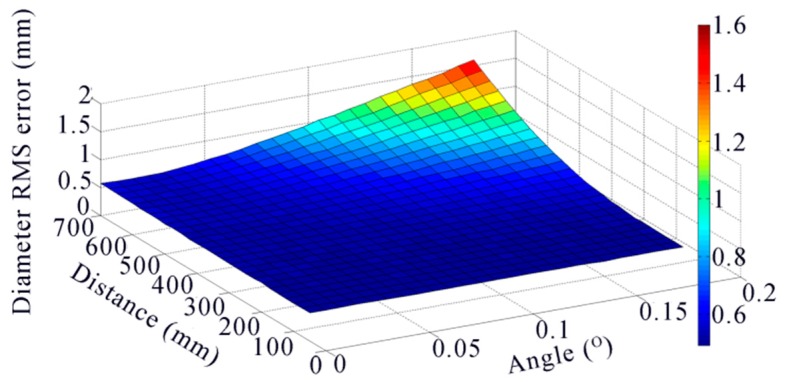
Diameter RMS error to the angle of bending and the distance from ***O1*** to the rim plane.

According to the European Norm EN 13103 [20], the finite element analysis of the wheelset shows that the maximum deformation appears at the middle, which is about 1 mm according to the different types and different materials of the wheelset. The distance between the two wheel rim planes is always normal, 1353 mm. Therefore, in the condition of the max deformation, the curvature radius of the axis is 0.229 km, and the angle between the two wheel rim planes is 0.34°. In the simulation, we change the angle between the two rim planes from 0.0° to 0.34° at the interval of 0.02°. What is more, the distance from the laser profile on the axle to the rim plane also influences the error of the wheel center. Therefore, we change the distance from 50 mm to 700 mm at the interval of 50 mm at the same time. For each change, 10,000 independent trials are performed, and the 3D reconstruction noise level is set to 0.2 mm. The result is shown as Figure 15.

**Figure 16:**
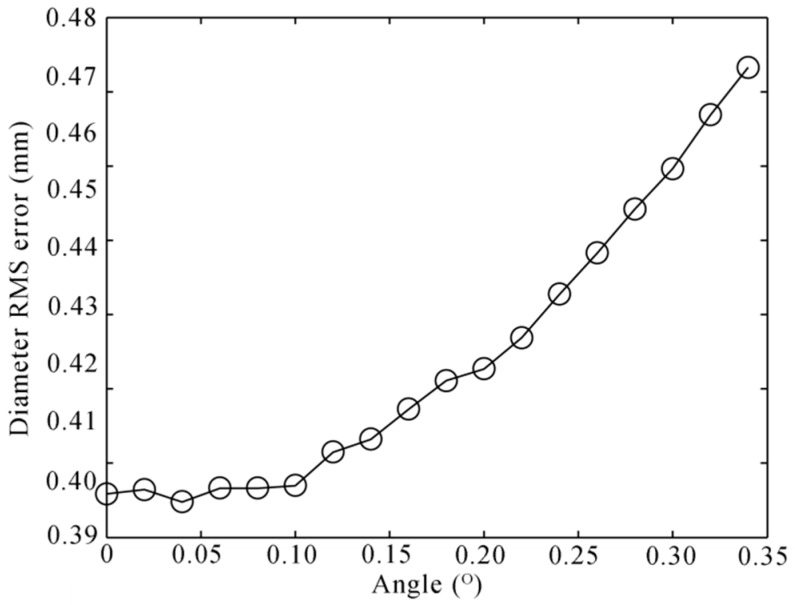
Diameter RMS error to the angle of the bend with the distance dL of 200 mm.

The diameter RMS error is growing with the angle and the distance. However, when the distance is shorter than 500 mm, the error almost does not change. What is more, the laser profile on the axle is easily blocked by the surroundings when it is too close to the wheel. We choice the distance of 200 mm that is most appropriate in our experience. We perform another simulation with this distance. The noise level is set to 0.2 mm, and 100,000 independent trials are performed. The result is shown in Figure 16.

### 3.3. The Factor of the Geometrical Error of the Wheelset Axle

**Figure 17:**
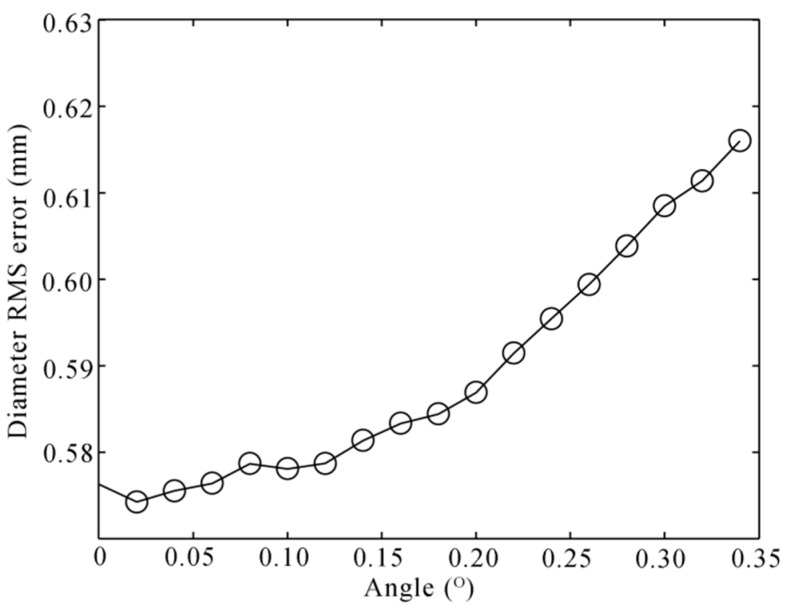
Diameter RMS error to the angle of bending with the geometrical error.

According to the European Norm EN 13261 [21], the geometrical tolerance of the axle body is 0.5 mm. We add zero mean noises of which the max is 0.5 mm to the 3D coordinates of the laser profile on the axle. The 3D reconstruction noise level is set to 0.2 mm, and the angle of bend changes from 0.0° to 0.34° at the interval of 0.02°. The result is shown in Figure 17. The diameter RMS errors increase about 0.15 mm compared to Figure 16.

## 4. Real Experiment

We performed both the static experiment and the dynamic field experiment. The static one was performed in the laboratory that had real rail tracks and two half wheels from a real wheel. The static experiment platform was the same as the one in our previous work [12]. The dynamic one was performed at an actual railway where trains were running normally.

### 4.1. Static Experiment

**Figure 18:**
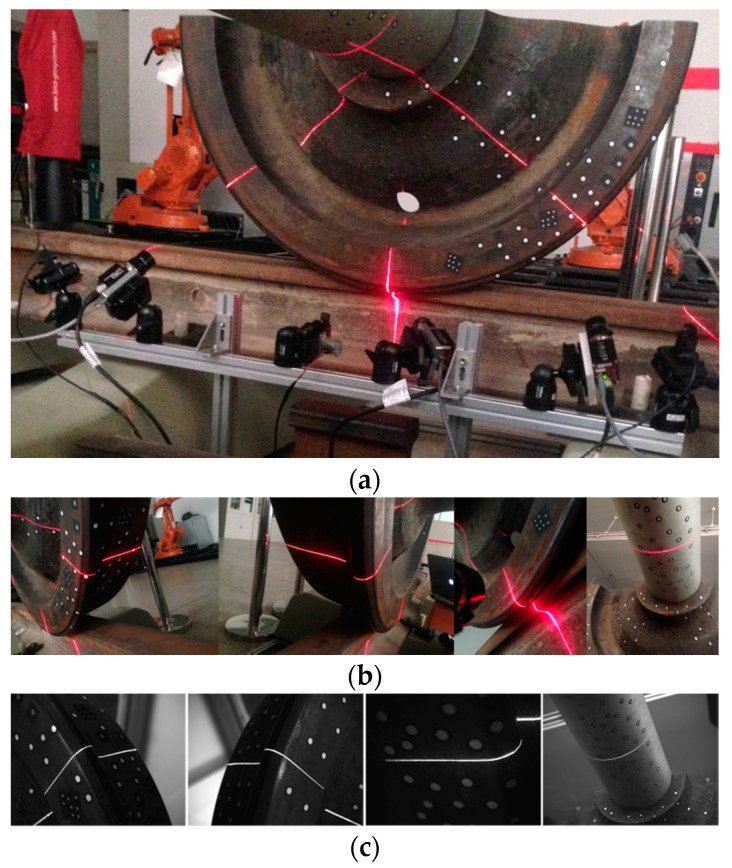
The static experiment. (**a**) The experiment platform; (**b**) the laser profiles on the wheelset; (**c**) the laser images captured by the cameras.

The static experiment platform and the laser images are shown in Figure 18. We used two half wheels and a tailor-made axle to simulate the wheelset. The structured-light sensors were composed of AVT GC1380H industrial 1380 × 1024 pixel cameras and 0.5-mW power, 808-nm wavelength line lasers. Because the wheel was not a standard unworked wheel, its 3D shape needed to be measured, so that we could get the truth-value of the diameter. We used the high-precision 3D scanning equipment called Creaform EXAscan, whose accuracy was 0.05 mm. It was also used for the global calibration of the structured-light sensors, as described in Section 2.3. The 3D shape of the wheel was scanned, and the diameter was calculated as 799.87 mm.

**Figure 19:**
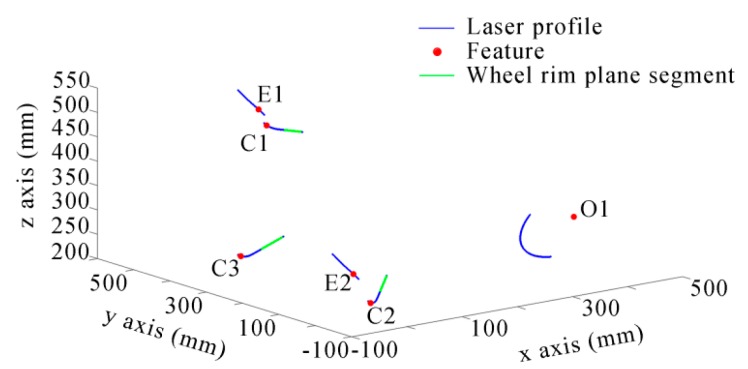
The 3D laser profiles.

**Figure 20:**
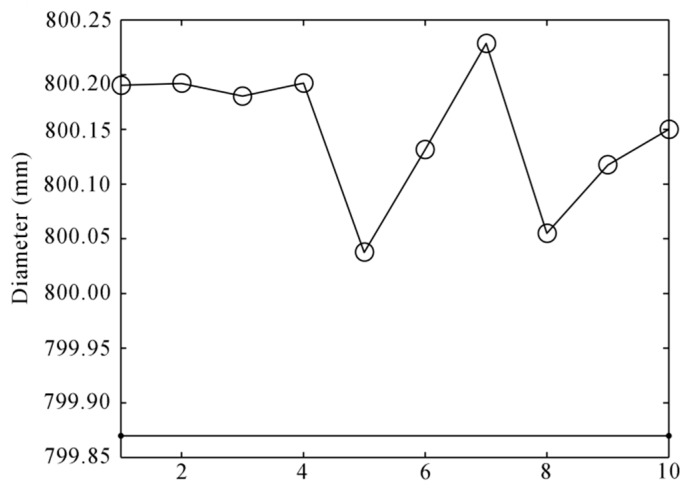
The diameter results of ten static measurements.

After the image processing and the 3D reconstruction of the structured-light sensors, the 3D profiles are shown in Figure 19. The equation of the rim plane and the features’ 3D coordinates are shown in Table 1. The ***E1***, ***E2*** and ***C1***, ***C2***, ***C3*** were extracted from the related laser profiles. ***O1*** was the center of the circle that was fitted using the laser profile on the wheelset axle. The rim plane was fitted using the three laser profiles on the wheel rim plane, and its fitting error was the RMS distance from all of the points of the laser profiles to the plane equation. ***O_o_*** was the result of the optimization using Equation (10), and its fitting error was the value of Equation (10) after optimization. The result of wheel diameter is shown in Table 2. We performed the static measurement ten times. The results are shown in Figure 20. The ambient light was very good and stable in the laboratory, so the noise was little. The measurement error was mainly derived from the calibration and the center position error of the laser stripe. The result changed a little. The static RMS error was 0.28 mm.

### 4.2. Dynamic Field Experiment

**Figure 21:**
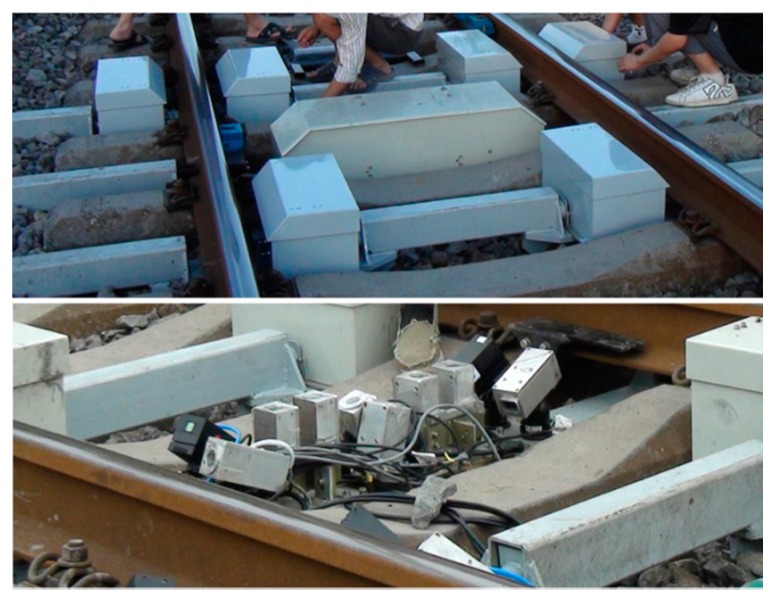
Wheelset dynamic measurement system installed on the railway bed.

The dynamic field experiment was performed at an actual railway in Gedian detection station, Wuhan City. This railway is one section of the railway line Wuchang to Jiujiang of China, in which the train is running at a speed of about 60 to 90 km/h. The wheelset measurement system was installed on the railway bed, as shown in Figure 21. The structured-light sensors were composed of AVT GC1380H industrial 1380 × 1024 pixel cameras and 4-W power, 808-nm wavelength line lasers. In order to avoid the vibration transmitted to the sensors from the rail, we designed the support mechanical structure that was not connected to the rail. The sensors were all installed in the boxes. The boxes were installed on the steel beam, which was supported at the two ends by the reinforced concrete deeply buried under the railway bed. Therefore, the boxes would not vibrate with the rail. Only the vibration from the concrete, which was very slight, could be transmitted to the sensors. This system had been continually used for three months, which proved reliable and secure for actual applications.

**Figure 22:**
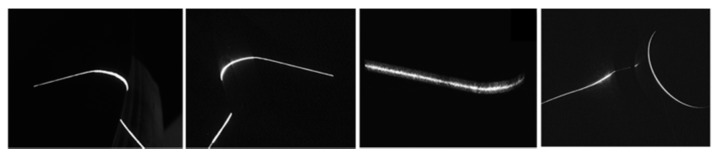
One set of laser images of a wheel.

The electromagnetic sensor would send a signal to make the sensors project lasers and capture the laser images when the wheelset arrived. Figure 22 shows one set of the laser images of a wheel. Then, the computer performed the image processing and the 3D reconstruction and then calculated the diameter.

**Figure 23:**
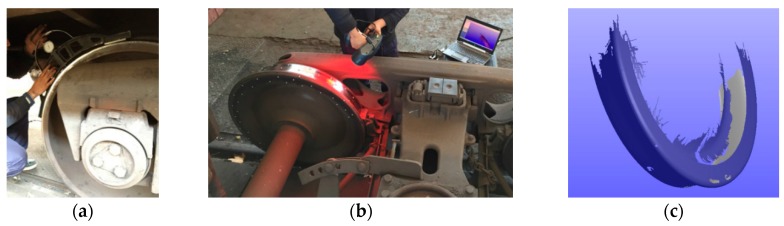
The manual measurement. (**a**) Using the diameter caliper; (**b**) using the 3D scanner; (**c**) the 3D shape result of the scanner.

We used the wheel diameter caliper to measure 16 wheels of two freight vehicles when these vehicles were transported to the train yard for maintenance. Each vehicle had its unique ID, which could help us to match the wheel with its diameter result of the measurement system. However, the resolution of the diameter caliper was 0.1 mm, and the operation skill influenced the result seriously. In order to get a high precision diameter, we also used the 3D scanner Creaform EXAscan to measure the first two wheels’ 3D shapes (the scanning work was very time consuming, so that we could not scan all of the wheels). The manual measurement is shown as Figure 23.

**Figure 24:**
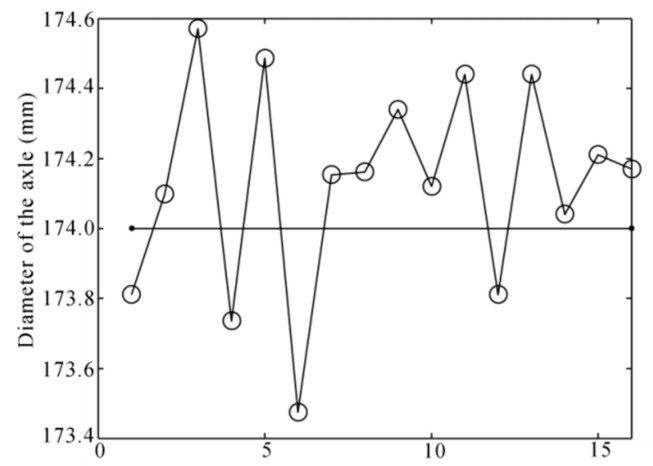
The diameter results of the wheelset axle.

Figure 24 shows the diameters of the 16 wheelset axle bodies that were measured. According to the results, the type of the wheelset axle could be determined, and the design size of its diameter should be 174 mm according to the Chinese Standard [22]. The difference between the measurement result and design size could give the geometrical error of the wheelset axle, which was about 0.32 mm.

**Figure 25:**
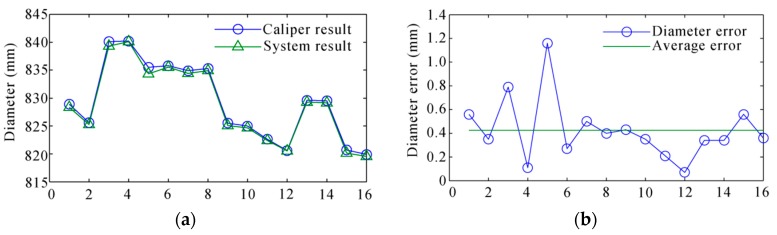
The comparison between the system result and the diameter caliper. (**a**) The diameter result; (**b**) the diameter error.

The comparison between the measurement result and the diameter caliper is shown in Figure 25. The average error was 0.43 mm with the standard deviation of 0.26 mm, and the RMS error was 0.50 mm. The result of the scanner is shown in Table 3. It showed that the system was very accurate and robust for field applications.

## 5. Conclusions

We propose a wheel diameter dynamic measurement method based on structured-light vision. It utilizes the wheelset axle to determine the wheel center position. Compared to the traditional method using the wheel tread only, it is more robust against noise. The flexible calibration method for the structured-light sensors is introduced, and the image processing method that can be adapted to any width and the gray level light stripe is introduced. These methods are very flexible and effective in our field use. According to the measurement purpose and environment, we select a set of configurations for the structured-light vision system, and the layout of the sensors is determined by the simulations. The real experiment in the actual railway showed that the method was very stable, and the measurement RMS error was less than 0.5 mm, which satisfied the requirement by the railway operation. This method is also available for the train that runs at the speed of about 80 km/h.

However, we have to point out that this method is mainly suitable for a freight train that has an exposed wheelset axle. For other types of trains, the wheelset possibly has big brake discs on the axle, which could block the laser and the view of the camera. The powered wheelset axle cannot use this method either, since the axle is surrounded by an electrical motor. Fortunately, the significance for the freight train is the most important, because its load and length is the biggest. For other trains, the laser displacement sensor or other measurement means, which can take the place of the structured-light sensor to measure the wheelset axle, can be attempted.

## Figures and Tables

**Table 1:** Results of the features and the wheel rim plane (in millimeters).

Feature	Coordinate/Equation	Fitting Error
E1	(148.31, 535.75, 471.05)	/
E2	(−31.09, −0.84, 291.13)	/
C1	(181.36, 565.43, 425.41)	/
C2	(−11.36, −13.69, 232.34)	/
C3	(−12.85, 343.45, 246.90)	/
O1	(477.62, 165.72, 267.44)	/
Rim plane	0.65345x + 0.035194y − 0.756148z − 170.55372 = 0	0.0031
O_o_	(290.51, 155.71, 483.85)	0.010

**Table 2:** Diameter result of the static experiment (in millimeters).

Measurement Result	True-Value	Error
800.15	799.87	0.28

**Table 3:** Wheel diameter results of the 3D scanner and the system (in millimeters).

Vehicle ID (Speed)	Wheel Num.	3D Scanner Result	System Result	Error
4840409 (76 km/h)	1	828.68	828.34	0.34
	2	825.38	825.25	0.13

## Abbreviations

The following abbreviations are used in this manuscript:

LDS	laser displacement sensor
CCD	charge coupled device
CCF	camera coordinate frame
ICF	image coordinate frame
WCF	world coordinate frame
FOV	field of view
RMS	root mean square
